# Identification of a Novel Dual Inhibitor of Acetylcholinesterase and Butyrylcholinesterase: In Vitro and In Silico Studies

**DOI:** 10.3390/ph16010095

**Published:** 2023-01-09

**Authors:** Raquel B. M. de Almeida, Deyse B. Barbosa, Mayra R. do Bomfim, Jéssika A. O. Amparo, Bruno S. Andrade, Silvia L. Costa, Joaquín M. Campos, Jorddy N. Cruz, Cleydson B. R. Santos, Franco H. A. Leite, Mariana B. Botura

**Affiliations:** 1 Department of Health, State University of Feira de Santana, Feira de Santana 44036-900, BA, Brazil; 2 Institute of Health Sciences, Federal University of Bahia, Salvador 40170-110, BA, Brazil; 3 Department of Biological Sciences, State University of Southwest of Bahia, Jequié 45208-091, BA, Brazil; 4 Biosanitary Institute of Granada (ibs.GRANADA), University of Granada, 18071 Granada, Spain; 5 Department of Pharmaceutical and Organic Chemistry, Faculty of Pharmacy, Campus of Cartuja, University of Granada, 18071 Granada, Spain; 6 Laboratory of Modeling and Computational Chemistry, Department of Biological and Health Sciences, Federal University of Amapá, Macapá 68902-280, AP, Brazil

**Keywords:** Alzheimer’s disease, acetylcholinesterase, butyrylcholinesterase, inhibitors

## Abstract

The enhancement of cholinergic functions via acetylcholinesterase (AChE) and butyrylcholinesterase (BuChE) inhibition is considered a valuable therapeutic strategy for the treatment of Alzheimer’s disease. This study aimed to evaluate the in vitro effect of ZINC390718, previously filtered using computational approaches, on both cholinesterases and to characterize, using a molecular dynamics (MD) simulation, the possible binding mode of this compound inside the cholinesterase enzymes. The in vitro cytotoxicity effect was also investigated using a primary astrocyte-enriched glial cell culture. ZINC390718 presented in vitro dual inhibitory activity against AChE at a high micromolar range (IC_50_ = 543.8 µM) and against BuChE (IC_50_ = 241.1 µM) in a concentration-dependent manner, with greater activity against BuChE. The MD simulation revealed that ZINC390718 performed important hydrophobic and H-bond interactions with the catalytic residue sites on both targets. The residues that promoted the hydrophobic interactions and H-bonding in the AChE target were Leu67, Trp86, Phe123, Tyr124, Ser293, Phe295, and Tyr341, and on the BuChE target, they were Asp70, Tyr332, Tyr128, Ile442, Trp82, and Glu197. The cytotoxic effect of Z390718, evaluated via cell viability, showed that the molecule has low in vitro toxicity. The in vitro and in silico results indicate that ZINC390718 can be used as chemotype for the optimization and identification of new dual cholinesterase inhibitors.

## 1. Introduction

Alzheimer’s disease (AD) is a neurodegenerative and irreversible disorder, characterized by a progressive decrease in memory and other cognitive functions that mainly affect the patient’s social life [1]. AD is the most common cause of dementia, especially in the elderly, who account for about 60% to 70% of cases [2]. AD has a complex and multifactorial etiology that involves low levels of acetylcholine, deposits of amyloid plaques, and neurofibrillary tangles of aggregated tau proteins in the brain [3]. 

Cognitive and behavioral disorders in AD are related to the rupture of cholinergic pathways in the cerebral cortex and in the basal forebrain; they are also associated with decreased levels of the neurotransmitter acetylcholine (Ach) [4]. In this context, studies on AD have shown abnormalities in many neurotransmission systems, and the most prominent and serious damage is that of the cholinergic system, with a selective loss of presynaptic cholinergic neurons that project in the cerebral cortex and hippocampus, leading to the cholinergic hypothesis of the development of AD [5]. 

Thus, the main approach to treat the disease is to restore the level of Ach through the inhibition of cholinesterase [6]. Cholinesterases are a group of esterase enzymes, and they are responsible for catalyzing the hydrolysis of Ach in choline and acetic acid and are divided into two types: acetylcholinesterase (AChE, EC 3.1.1.7) and butyrylcholinesterase (BuChE, EC 3.1.1.8) [7]. They are homologous glycoproteins and share 50% of sequential identity, but they differ in their distribution in tissues, in their kinetic properties, and in the specificity of their substrates [8].

AChE is found abundantly in the central nervous system (CNS) and is expressed by neurons in skeletal muscles and the erythrocyte membrane, while BuChE is associated with glial cells and is found mainly in blood plasma [8]. Cholinesterase inhibitors, recognized as an official treatment of Alzheimer’s dementia, are also prescribed for dementia without Alzheimer’s; psychiatric disorders; and neurological diseases, such as Huntington’s disease (HD), cerebral autosomal dominant arteriopathy with subcortical infarcts and leukoencephalopathy (CADASIL), frontotemporal dementia (FTD), dementia in multiple sclerosis (MS), and progressive supranuclear palsy (PSP) [9].

During the development of AD, AChE levels in the CNS decrease, while BuChE activity increases progressively and, consequently, mainly BuChE controls Ach regulation. In addition, studies employing an AD mouse model have suggested that BuChE may play a role in the etiology of AD due to its contribution to the deposition of amyloid plaques [10]. Thus, an agent that inhibits both AChE and BuChE (a dual inhibitor), such as rivastigmine, provides greater benefits to patients with dementia by decreasing the compensatory effect of Ach hydrolysis by BuChE when AChE levels decrease [11].

Currently, the FDA-approved anticholinesterase drugs are donepezil and galantamine (AChE inhibitors) and rivastigmine (dual inhibitor), which have beneficial effects on the cognitive, functional, and behavioral symptoms of the disease [12]. These drugs are generally well-tolerated, but symptoms such as diarrhea, nausea, vomiting, muscle cramps, decreased blood pressure, insomnia, fatigue, and a loss of appetite have been reported [13]. 

In this context, much effort has been directed towards the development of new dual cholinesterase inhibitors for the treatment of AD neurological disorders [14]. A combination of in silico and in vitro studies represents a valuable strategy to find new candidate drugs targeting different enzymes related to AD [15].

The toxicity evaluation of bioactive molecules is an important step in the development of drugs. In vitro cytotoxicity assays with cultured cells are widely used for preliminary toxicological studies of chemicals and for drug screening [16,17,18]. For substances with action on the central nervous system, evaluations of cytotoxic effects on neural and glial cells are performed. Astrocyte cultures can be used to evaluate compounds for neurotoxicity or the ability of these compounds to protect astrocytes from disease [19].

Thus, the aim of this study was to identify a new molecule with potential dual inhibition against acetylcholinesterase and butyrylcholinesterase using in silico tools and evaluations of enzymatic inhibition and preliminary in vitro toxicity.

## 2. Results and Discussion

### 2.1. In Vitro Anticholinesterase Assays

#### 2.1.1. Kinetic Parameters

For enzymatic assays, it must be considered that the reactions of the enzymes also depend on factors such as the pH of the substrate and enzyme. The optimal pH of many enzymes is within the physiological range, often between pH 7 and 8 [20]. The normal arterial pH of humans is 7.4, and the blood pH of some other species differs slightly from pH 7.4 [21]. It has been observed that the maximum cholinesterase catalytic range occurs at pH 7.4. 

**Figure 1:**
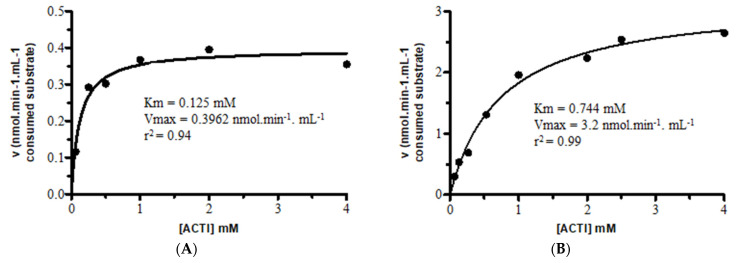
Determination of the kinetic parameters of AChE and BuChE under steady-state conditions. (**A**) Km and Vmax values of AChE against the substrate (ACTI). (**B**) Km and Vmax values of BuChE against the substrate (ACTI). Values were calculated using non-linear regression.

The concentration of the substrate used in kinetic tests directly influences the chance of identifying inhibitors [22]. The Km and Vmax values of the substrate used in the enzymatic reactions with the cholinesterases were determined, as shown in Figure 1.

The concentration of the DTNB followed the value described by Ellman et al. [23] (0.01 M). Even when carrying out tests with varying concentrations, it was observed that this concentration could be maintained without interfering with the reaction of the enzymes. The reading times of the enzymatic reactions with AChE (10 min) and BuChE (20 min) were determined after identifying the decline in the line that represents the end of the total substrate consumption rate.

#### 2.1.2. Dual Anticholinesterase Activity of ZINC390718

**Figure 2:**
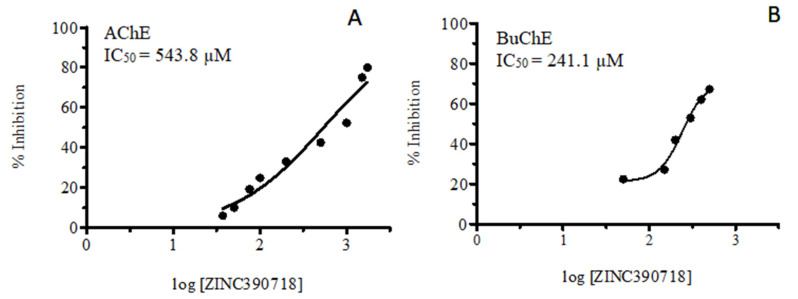
Concentration–response curve of the in vitro anticholinesterase effect of ZINC390718: (**A**) acetylcholinesterase (AChE) and (**B**) butyrylcholinesterase (BuChE).

In an attempt to assess the ability of ZINC390718 to inhibit the two enzymes, the IC_50_ values of both targets were calculated (Figure 2). After kinetic parametrization, the enzymatic inhibition activities of the compound ZINC390718 against AChE and BuChE showed a significant effect (*p* < 0.05) in a concentration-dependent manner for both enzymes (Figure 2). The IC_50_ concentrations found were 543.8 and 241.1 µM for AChE and BuChE, respectively.

ZINC390718 showed a greater selectivity for BuChE than AChE (selectivity index = 2.25), although for both targets, high IC_50_ concentration values were observed. In AChE, the estimated volume of the active site is relatively small (302 Å^3^), being coated with fourteen aromatic amino acid residues, while that of BuChE, with only eight aryl residues, is considerably higher (502 Å^3^), and this difference in volume can influence the determination of the size of the inhibitor molecules and their accommodation in the active site of each enzyme, thus providing a means of selectivity for the inhibitor [24].

**Figure 3:**
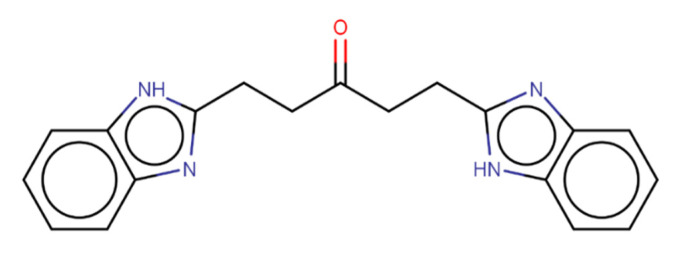
The 2D chemical structure of ZINC390718, C_19_H_18_N_4_O, with nitrogen groups (blue) and carbonyl (red) highlighted.

ZINC390718 is a symmetric molecule characterized by the presence of nitrogen groups linked to two rings, as shown in Figure 3. A class of organic compounds with potential anticholinesterase activity is alkaloids, which also contain nitrogen bases as one of their main characteristics [25]. 

The benzimidazole radical (a benzene fused to the pentagonal ring) present at the ends of ZINC390718 gives it an association with imidazole alkaloids, a group that exhibits many pharmacological activities [26]. The research carried out by Gurjar et al. [27] shows the imidazole analogues as potential cholinesterase inhibitors with a neuroprotective role for Alzheimer’s disease. Hence, ZINC390718 represents a promising bioactive molecule, and future research aiming to improve its molecular arrangement could potentially make it more active against its biological targets.

### 2.2. In Vitro Neurotoxic Effects 

**Figure 4:**
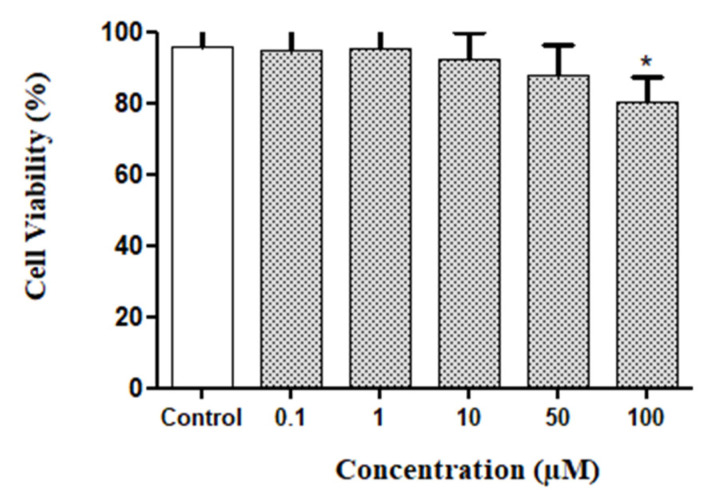
Percentage of cell viability in glial cell cultures of rats after 24 h of exposure to the Z90718 molecule (MTT assay). (*) Significant differences from negative control (*p* < 0.05).

The ZINC390718 molecule was subjected to a primary assessment of its toxicity against glial cells and astrocytes, and the results are shown in Figure 4. Cell viability analyses, carried out by measuring the function of mitochondrial dehydrogenases in converting MTT into crystals of formazan, allowed for the interpretation that, after 24 h of exposure, the study molecule (ZINC390718) had a concentration-dependent effect. The statistically significant interference with cell viability occurred only at the highest concentration tested (100 µM). 

The percentage of viable cells was proven to be high for the other concentrations (between 88 and 95%), considering the percentage of the negative control (95%), which suggests a low toxicity. The MTT assay investigates whether cells are metabolically active to provide their dehydrogenase with enough reduced NADH/NADPH cofactors to reduce MTT to blue formazan [28].

In the analysis of the cultures treated with the molecule under study by using phase-contrast microscopy, no changes in the morphology of the treated cells, at different concentrations in relation to the negative control, were identified.

The concentrations evaluated in the toxicity test were lower than the values found for IC_50_ in the enzymatic assays with the cholinesterases. However, for this cell culture model, the concentration of 100 mM can be considered high. Higher concentrations can interfere with the culture medium and, consequently, cell viability.

Other studies have also used astrocyte cultures to assess the potential for chemical cytotoxicity. In a previous study, an assay was used to test a library with 80 compounds in MTT, and 4 already known cytotoxic compounds (valinomycin; 3,3′, 5,5′-tetrabromobisphenol; deltamethrin; and triphenyl phosphate) were used as controls in populations of neural cells derived from induced pluripotent stem cells (iPSCs) (neuroepithelial stem cells (NSCs), neurons, and astrocytes) at concentrations of 1, 10, and 100 µM [29]. Although 1 µM did not indicate the cytotoxicity of any of the tested compounds already known to be cytotoxic, at a concentration of 10 µM, it was possible to observe a cytotoxicity profile and the loss of cell viability caused, for example, by deltamethrin.

Historically, drug development programs for neurodegenerative diseases generally only target neurons, ignoring the contributions of astrocytes and instead targeting them, offering a new approach to drug development for the treatment of neurological diseases [30]. Preliminary tests on cells of great importance, such as glial cells, for the identification of the adequate functional capacity of the central nervous system, including neurodegenerative diseases, can lead to future evaluations that may bring more conclusive results from the perspective of the cytotoxicity of potentially active molecules in biological targets.

### 2.3. Molecular Dynamic Simulations

**Figure 5:**
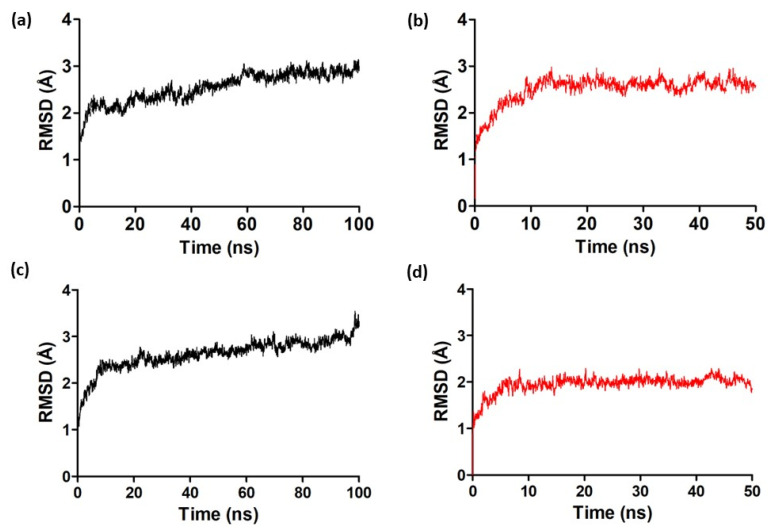
RMSD (backbone) of the APO structures and AChE and BuChE complexed with ZINC390718 during molecular dynamics of 100 ns (APO forms) and 50 ns (complexed forms). (**a**) AChE APO form; (**b**) AChE complexed with ZINC390718; (**c**) BuChE APO form; (**d**) BuChE complexed with ZINC390718.

The molecular dynamics (MD) simulations allowed us to observe the stability and patterns of the established interactions of ZINC390718 with the active sites of the enzymes AChE and BuChE. To ensure reliable data acquisition, one needs to ensure that the system is stable, and one of the main ways to assess structural stability is by using RMSD graphs. This metric is used to analyze the equilibrium and stabilization of the protein structure and its system during the trajectory of MD simulations [31,32,33]. The complexes (ZINC90718 bound to AChE and BuChE) and the APO forms of the proteins were obtained from the RMSD for the main chain (backbone), along with the trajectory (see Figure 5).

In MD simulations, evaluations of the graphs allow for observations of progressive increases in the RMSD values of a protein until the values stabilize. The stability of AChE in the APO form (enzyme without the ligand) was observed from 60 ns (the production phase) of the simulation, where the mean RMSD value was 2.5 ± 0.3 Å; as the enzyme complexed with the ligand (ZINC390718), its production phase was from 15 ns, and the average RMSD value was 2.5 ± 0.3 Å. The stability of BuChE in the APO form was observed from 20 to 95 ns of the simulation, where the mean RMSD value was 2.6 ± 0.3 Å; as the enzyme complexed with the ligand (ZINC390718), its productive phase was 5 ns, and the mean RMSD value was 1.9 ± 0.19 Å. 

An RMSD lower than 3.0 Å is considered appropriate when all atoms of the system are used in the evaluation, considering that the equilibrium variation occurs according to the intrinsic characteristics of the protein. The conformational changes that occurred in the AChE and BuChE structures in relation to the initial structures and the identification of the moment when the systems reached equilibrium were also analyzed using the RMSD metric, which mathematically suggests subtle changes between the patterns of the enzymatic systems presented by the APO forms and complexed with the ZINC390718 molecule when compared to each other [34].

**Figure 6:**
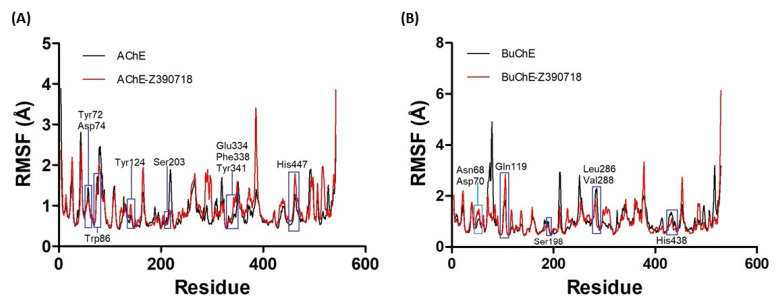
RMSF (Å) of backbone Cα atoms of the APO structures and complexes of AChE (**A**) and BuChE (**B**) with ZINC390718 during the production phase (AChE apo: 15 to 100 ns; AChE complex: 15 to 50 ns) (BuChE apo: 20 to 95 ns; BuChE complex: 5 to 50 ns). Highlights in blue correspond to regions of the active site.

The RMSD data are not enough to guarantee the stability of a system, as they do not score the variations in certain regions of the structure. Therefore, an RMSF plot was also generated in an attempt to evaluate the fluctuations of the atoms of each residue during the trajectory. Thus, waste fluctuation graphs were generated for the APO form and compared with those generated for the respective complexes with ZINC390718 during the production phase (Figure 6).

The analysis of the RMSF values demonstrated the atomic fluctuations of the APO form of AChE (RMSF = 0.8 ± 0.4 Å) and its complex (RMSF = 0.9 ± 0.4 Å), as well as the APO form of BuChE (RMSF = 1.0 ± 0.5 Å) and its complex (RMSF = 0.9 ± 0.50 Å). Comparing the regions with the greatest fluctuations between the APO and complex systems (Figure 6), it was observed that the stability pattern remained approximate, with statistically non-significant variations in the values.

The divergences in the findings between AChE and BuChE can be explained by differences in the structures around the main entrance area of the enzyme. In AChE, there are eight aromatic residues: three of them are located at the entrance to the active site (Tyr 72, Trp 286, and Tyr 341) and five are located at nearly the same depth within the slit (Tyr 124, Phe 295, Phe 297, Tyr 337, and Phe 338). 

In BuChE, however, six of these residues are altered and substituted for minor residues; that is, Tyr 72 is substituted for Asn 68, Tyr 124 is substituted for Gln 119, Trp 286 is substituted for Ala 277, Phe 295 is substituted for Leu 286, Phe 297 is substituted for Val 288, and Tyr 337 is substituted for Ala 328. These smaller residues significantly extend the entry radius, which makes it possible for large substrates to enter the active site [35].

The data presented herein indicate that the presence of ZINC390718 possibly stabilizes the structures of AChE and BuChE. However, in order to understand the stabilization of the systems, we investigate the intermolecular interactions related to the processes that likely contribute to obtaining a biological response. Thus, hydrogen bonds stand out, since they correspond to the main interactions involved in maintaining the tertiary structure and protein folding, as well as in molecular recognition [36].

Hydrogen interactions have transient characteristics, making it necessary to evaluate their occurrence and permanence during the simulation time. In order to identify whether the stability of the systems, the objective of this study, occurs due to these interactions, Table 1 shows the number and permanence of H bonds formed between the ligand ZINC390718 and the amino acids of the active sites of AChE and BuChE.

The residues that H-bonded with AChE with a permanence greater than 10% were Tyr124 with N1 (16.52%) and N2 (23.36%), Phe295 with O1 (86.61%), Arg296 with O1 (21.37%), and Ser293 with N4 (55.84%). Based on these data, the interactions of the H bond with the amino acids stand out in terms of their relationship with AChE’s active sites. The residue Tyr124 participates in the anionic peripheral active site, and Phe295 is part of the acyl pocket and presents a H interaction with a longer residence time. The residues Tyr124 and Phe295 perform interactions with cannabinoids and molecules that inhibit the enzymatic activity of AChE [37].

AChE also has other important binding sites for ligand-receptor mechanisms: the peripheral anionic site (PAS) (Tyr103, Asp105, Tyr155, Glu316, Trp317, and Tyr372), the acyl pocket (Trp117), the anionic site (Trp267, Phre26, and Phe328), the oxyanionic orifice (Gly152, Gly153, and Ala235), and the aromatic site (Tyr164 and Tyr368) [38].

The interactions of H with BuChE, which remained greater than 10%, were observed with the residues Asp70 (37.9%), Glu197 (12.4%), Tyr332 (38.4%), and Lis339 (33.0%). Asp70 and Tyr332 are residues located at the peripheral site of BuChE; these residues interact with the substrate and change the conformation of the protein to allow for catalysis [39].

The residue Tyr332 has been implicated in binding substrates to BuChE, suggesting that this amino acid residue is part of the P (peripheral) site in this enzyme; anionic Asp70 (Asp74 in AChE) is linked by H interactions, and Tyr332 is the other essential residue of the P site of BuChE [40].

The AChE and BuChE cavities resemble a deep, narrow canyon, about 20 Å long in both enzymes. The catalytic triad consists of serine, histidine, and glutamate residues. The peripheral site that influences the catalytic process is located above the catalytic triad towards the periphery of the active site canyon. This peripheral site contains several aromatic amino acid residues, as well as the aspartate residue (Asp70), an amino acid from BuChE that has been found to have one of the highest percentages of permanence in interactions with H [38].

To select the representative structures of the systems, the most adequate RMSD values were 1.2 Å (AChE) and 1.3 Å (BuChE), as they exhibited the largest number of clusters and significant diversity, and of the total system conformations during the production phase, more than 50% presented in the first three groups for the three systems [41]. The representative structures selected were those that were the most frequent in the groups with the highest number of conformations observed for the cutoff point.

The cutoff point was defined based on the concept that the greater the number of clusters observed in a simulation, the greater the diversity of the conformations adopted by the complex. Moreover, the representative structures were selected via the evaluation of the clusters, using the differences in the maximum RMSD values among the conformations to determine the cutoff point while still considering the native state of the protein as the most stable conformation, which certainly is the most frequent and the most populous group [42].

**Figure 7:**
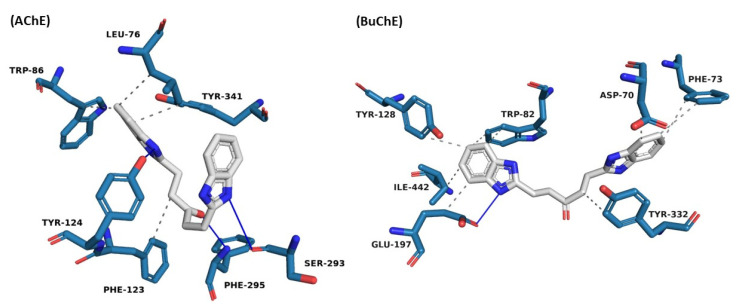
ZINC04541733 interactions at AChE and BuChE binding sites obtained from the representative structure of the MD simulation. White: ligand carbons; blue: nitrogen; red: oxygen; white sphere: aromatic center; solid blue line: hydrogen bond; gray dashed line: hydrophobic interaction.

The representative structures below were selected based on their frequency in the groups with the highest number of conformations observed for the cutoff point (AChE: 26.5 ns; BuChE: 39.15 ns). The set of intermolecular interactions generated between the targets (AChE and BuChE) and the ligand (ZINC390718) responsible for the stabilization of the systems was illustrated using PLIP (Figure 7) after identifying the representative structures for the complexes.

When evaluating the representative structure of the ZINC390718 complex with AChE and BuChE, hydrophobic and hydrogen interactions are observed. The ligand has a benzimidazole radical (a benzene fused to the pentagonal ring) at each of its ends. In the interactions of AChE with the ligand, one of the benzimidazole radicals is involved in the hydrophobic interactions with the residues Leu76, Trp86, and Tyr341, and the N atom establishes a hydrogen interaction with Tyr124. 

However, the N opposite to the benzimidazole radical performs a hydrogen interaction with Ser293. Additionally, a hydrophobic interaction is observed between the linker chain and Phe123.

The attractive and non-covalent interactions between the ligand with the residues Tyr 341 and Trp 86 play an important role in the stabilization of the inhibitor in the active site [43]. The peripheral site of AChE is composed of numerous aromatic chains that extend beyond the Tyr337 residue at the catalytic site interface to the entrance of the canyon, contributing to the catalytic efficiency of AChE. This site includes Phe295, Tyr337, and Tyr341 [44].

One end of the region of the linker, the benzimidazole radical, interacts with the Asp70 and Tyr332 residues of BuChE through hydrophobic interactions. The other end, which is also formed by the same benzimidazole radical, interacts hydrophobically with the residues Tyr128, Ile442, Trp82, and Glu197, the latter also interacting with hydrogen as a donor with one of the nitrogen atoms present in this radical.

It has been reported in the literature that the occurrence of hydrogen interactions with the Asp70 residue is important for inhibitory activity against BuChE, because it promotes the blocking of the active site and prevents substrate access to this region; consequently, catalysis does not occur [45].

The interaction with Trp82 (also observed in the structure obtained via molecular docking) is observed in the MD simulations with BuChE inhibitors, and this residue even interacts with the benzene ring of tacrine (cholinesterase inhibitor), playing a crucial role in stabilizing the substrate in the active site [46].

In the studies performed by Zhang et al. [47], the residues, including Asp70, Trp82, and Tyr332, were identified as playing important roles in stabilizing tacrine in the BuChE pocket, where important interactions, such as hydrogen interactions, were observed. Tyr332 and Asp70 residues, which act as gatekeepers, play crucial roles in substrate adjustment.

## 3. Materials and Methods

### 3.1. Reagents and Equipment

For the evaluations of anticholinesterase activities, acetylcholinesterase (AChE) (*Electrophorus electricus* type VI) and butyrylcholinesterase (BuChE obtained from equine serum), the substrate acetylthiocholine (ACTI), Ellman’s reagent, 5.5′ditiobis (2nitrobenzoic acid) (DTNB), bovine serum albumin (BSA), and the ZINC390718 molecule were obtained commercially from Sigma-Aldrich^®^. A Multiskan FC microplate reader was used and was obtained from Thermo Scientific^®^ (Waltham, MA, USA).

For the cytotoxicity assay, the following reagents were used: Dulbecco’s modified Eagle’s medium (DMEM, Cultilab, Campinas, SP, Brazil), penicillin, streptomycin, amphotericin B (Sigma-Aldrich^®^, São Paulo, SP, Brazil), and fetal bovine serum (SFB, Gibco, Grand Island, NY 12657, USA). The 3-(4,5-dimethylthiazol-2-yl)-2,5-diphenyltetrazolium (MTT) bromide reduction method was used (Sigma Aldrich, St. Louis, MO, USA).

The ZINC390718 molecule, evaluated in this study, was selected according to in silico data (pharmacophore-based virtual screening and a molecular docking analysis) obtained by our research group [15].

### 3.2. Determination of the Kinetic Parameters of AChE and BuChE

The standardization of the AChE and BuChE catalytic parameters was evaluated according to Ellman et al. [23] with modifications. The determination of the enzymatic concentration was carried out by keeping the concentration of the used substrate (acetylthiocholine: ACTI) fixed and by varying the enzyme concentrations (0.015, 0.075, and 0.30 U/mL) to evaluate the enzyme reaction rate and substrate consumption. The optical absorbance of the samples was quantified using a Multiskan FC microplate reader (Thermo Scientific^®^) at a wavelength of 405 nm and a temperature of 25 °C.

After the determination of the enzymatic concentrations, the Michaelis–Menten constant (Km value) for ACTI was determined by varying its concentration (0.0625; 0.1325; 0.2650; 0.5300; 1; 2; 4 mM) for each cholinesterase. The experiments were performed in triplicate, and the Km and Vmax values were obtained using non-linear regression (Michaelis–Menten equation), utilizing the GraphPad Prism program (version 5.0).

### 3.3. In Vitro Anticholinesterase Activity 

The evaluation of anticholinesterase activity was performed as described by Ellman et al. [23] adapted for 96-well microplates, using the kinetic parameters previously determined in Section 2.2.

Pipetting in all wells was initially carried out in the experiments using the following: 140 μL of 0.1 M phosphate buffer at pH 7.4 containing 0.1% bovine serum albumin (BSA); then, in separate triplicates, 20 μL of the sample (molecule ZINC390718 diluted in ethanol), positive control (0.5 mM eserine), and controls (10% ethanol and phosphate buffer); and 20 μL of enzyme in all triplicates (AchE: at a final concentration of 0.15 U/m and BuChE: at a concentration of 0.50 U/mL, both diluted in 0.1 M phosphate buffer). After a 10-minute incubation at room temperature (25 °C), 10 μL of DTNB (10 mM in the well) and 10 μL of ACTI (0.125 mM in the well for AChE, and 0.744 mM in the well for BuChE) were added. 

The sample (ZINC390718) was tested at concentrations between 37.5 and 1000 μM and 50 and 500 μM to calculate the IC_50_ values of AChE and BuChE, respectively. The readings were performed in a cycle from 0 to 11 per minute for AChE and in a cycle from 0 to 21 per minute for BuChE. The optical absorbance of the samples was measured at 405 nm using a Multiskan FC microplate reader (Thermo Scientific^®^).

### 3.4. In Vitro Evaluation of Neurotoxic Effects 

#### 3.4.1. Primary Astrocyte-Enriched Glial Cell Culture

The glial cell culture was obtained from the cortex of newborn Wistar rats, 0-2 days after their birth. The animals were acquired from the Vivarium of the School of Veterinary Medicine and Animal Sciences of the Federal University of Bahia (UFBA). The procedures were carried out in accordance with the Brazilian guidelines for the production, maintenance, and use of animals for teaching and scientific research activities of the Local Animal Experimentation Ethics Committee (CEUA) of the UFBA Institute of Health Sciences, approved under the registration no. 027/2012, composing the project entitled “Pharmacological bioprospecting of natural products and derivatives in cells of the central nervous system”. 

According to the protocol established by Pitanga et al. [48] and Silva et al. [49], glial cells were obtained from the beheading of the animals, followed by the removal of the brain hemispheres aseptically. The meninges and blood vessels were removed from each cortex, and then the material was mechanically dissociated and filtered through a sterile membrane (75 mm). The dissociated cells were grown in bottles with Dulbecco’s modified Eagle’s medium (DMEM), 50% glucose (33 mM), penicillin (100 IU/mL), streptomycin (100 μg/mL), NaHCO_3_ (2.438 g/L), 6.25 μg/mL amphotericin B, and 10% SFB. The cells were kept in a mixed glial culture for up to 7 days in culture bottles. The microglial cells were isolated from the mixed glial culture by shaking at 1000 RPM for 3 h at 37 °C. After the end of the agitation, the medium was collected, and, thus, the astrocyte-enriched glia remained adhered to the culture bottle. After trypsinization using a trypsin/EDTA solution (0.05% trypsin and 0.02% EDTA in PBS free of Ca^2+^/Mg^2+^) and counting in a Neubauer chamber, the cells were seeded at a density of 3.3 × 10^4^ in 96-well plates, previously treated with poly-D-lysine and cultured in a humid chamber with 5% CO_2_ at 37 °C.

#### 3.4.2. Analysis of Cytotoxicity through the MTT Assay

Cell viability was measured using the 3-(4,5-dimethylthiazol-2-yl)-2,5-diphenyltetrazolium (MTT) bromide reduction method according to Hansen et al. [50]. Cell cultures of normal glial cells were grown in 96-well culture plates, and after 24 h, they were exposed to the molecule in increasing concentrations (0.1, 1, 10, 50, and 100 µM) and to the control (DMSO 0.01%). The experiments were analyzed after 24 h of treatment. After the end of the treatment exposure time, the MTT solution (100 µL/well) diluted in DMEM, at a ratio of 1:4, was added. The plate containing the solution was kept incubated in an oven at 37 °C for 2 h, and then, for the lysis of the cells and complete dissolution of the formazan crystals, a volume of 100 µL/well of the lysis buffer was added, composed of 20% of sodium duodecyl sulfate (SDS) and 50% dimethyl formamide (DMF), pH 4.7.

The cultivation plate was kept at rest on the bench for 18 h, properly protected from light at 37 °C. After this time, the optical absorbance of each sample was quantified using a spectrophotometer (VARIOSKAN FLASH, Thermo Fisher Scientific) at a wavelength of 595 nm. An empty well for each treatment was used as a blank, and its absorbance value was discounted from all others. The experiments were performed in eight replicates, and the results are presented as the percentage of viability (mean and standard deviation) in relation to the control. Cell viability was calculated as a percentage of the negative control, which was considered to be 100%.

#### 3.4.3. Analysis of Cell Morphology Using Phase-Contrast Microscopy

The effects of the molecule on cell morphology were initially observed using phase-contrast microscopy. After 24 h of exposure to the molecule at different concentrations, the cell cultures were analyzed using phase-contrast microscopy with a green filter (Nikon TS-100), and then 3 fields were photographed for each treatment.

### 3.5. Statistical Analysis of In Vitro Tests

All generated data received adequate statistical treatment using GraphPad Prism software, version 5.0, for Windows (GraphPad Software, San Diego, CA, USA), and they are expressed as means ± standard deviation. The p values adopted as statistically significant in the analyses were those lower than 0.05. The IC_50_ values for anticholinestease activities were calculated using a non-linear regression analysis. 

The selectivity index (IS) of the molecule action against BuChE was determined using the following formula: IC_50_AChE/IC_50_*Bu*ChE.

### 3.6. In Silico Evaluation: Molecular Dynamics 

#### 3.6.1. Ligand Topology

The best pose of ZINC390718 obtained via the molecular docking assays was submitted to the ATB 3.0 server (https://atb.uq.edu.au/index.py, accessed on 5 July 2021) for the generation of its topology. The parameters of atomic charge, bond length, torsional angles, and dihedrals were obtained using the GROMOS96 54A7 force field [51].

#### 3.6.2. Molecular Dynamics Simulations

MD simulations were performed using the GROMACS 5.1.2 package [52], in which the GROMOS96 54A7 force field parameters, a temperature of 298.15 K, and a pressure of 1.013 bar were adopted.

The 3D structures were obtained from the PDB (PDB ID for AChE: 4M0E, and for BuChE: 4BDS), from which the crystallographic ligand, non-structural water molecules, and crystallization artifacts were removed. The non-modeled regions of the structures were built with the help of the SWISS-MODEL server [53].

The protonation states of the acidic and basic residues of the targets were adjusted in the pdb2gmx module implemented in GROMACS 5.1.2 according to pH 7.4 for AChE and BuChE [54]. For this, the pKa values of the residues were evaluated on the H ++ server (http://biophysics.cs.vt.edu/index.php, accessed on 5 July 2021) [55]. To solvate the systems, a dodecahedral box with the water model SPC-E [56] was used, with a minimum distance of 1.4 nm from the edge of the box. For neutralization, in systems involving AChE (APO and complex), seven Na2+ ions were added, while in systems with BuChE, four Cl− ions were added.

All systems (APO and complex forms) were minimized in two steps: initially by using the Steepest Descent (SD) algorithm with 10,000 cycles and, later, by using the Conjugated Gradient (GC) algorithm with 1000 cycles. After the minimization steps, the balancing step (t = 1.0 ns) and, finally, the production dynamics (APO systems: t = 100.0 ns; complex systems: t = 50.0 ns) were carried out. The MD data were obtained from the isothermal–isobaric ensemble (NPT—number of particles, constant temperature, and pressure).

The electrostatic and hydrophobic interactions were described using the Particle Mesh Ewald (PME) method [57], with a cutting radius equal to 0.9 nm. The stability of the system was evaluated based on the analysis of the variation in the Root Mean Square Deviation (RMSD) and the Root Mean Square Fluctuation (RMSF), calculated through the modules rms and rmsf available in GROMACS 5.1.2.50.

Additionally, the number and permanence of hydrogen bonds in the complexes were calculated using the g_hbond module, implemented in the GROMACS 5.1.2 package and the HbMap2Grace program (http://lmdm.biof.ufrj.br/software/hbmap2grace/index.html, accessed on 5 July 2021). The hydrogen bonds that showed a distance between the donor and acceptor that was lower than or equal to 3.5 Å and an angle between the donor/acceptor and the H atom that was lower than or equal to 60° were identified, as well as the bonds whose permanence time was lower than 10% of the total simulation time [58].

#### 3.6.3. Clustering Analysis and Selection of Average Structure

A representative structure of the ligand-macromolecule complex during the MD production phase was obtained by grouping similar conformations with the aid of the g_cluster module available in the GROMACS 5.1.2 package, using the joining method GROMOS [59], with cutoff points defined from the RMSD values of 1.0; 1.1; 1.2; 1.3; 1.4; 1.5 Å. The average structure of the most populous group was then selected for the analysis of the main interactions established between the prioritized ligand and the targets.

## 4. Conclusions

The ZINC390718 molecule had dual in vitro inhibitory activity against AChE and BuChE, acting in a concentration-dependent manner and being more active against BuChE. These in vitro effects are in agreement with the in silico findings. The MD simulation revealed that ZINC390718 performed important hydrophobic and H-interactions with residues that are part of AChE and BuChE catalytic sites. The residues Tyr341, Phe338, Tyr337, Val365, Val 294, Trp 86, Phe 295, and Gly342 participated in interactions with AChE, and the residues Asp70, Tyr332, Tyr128, Ile442, Trp82, and Glu197 participated in interactions with BuChE. These results support the ability of ZINC390718 to interact at the active sites of AChE and BuChE.

## Figures and Tables

**Table 1:** Permanence time of hydrogen interactions (H_bond_) in the active sites of AChE and BuChE during the production phase and identification of the pairs involved.

AChE				
Residue	Protein	ZINC390718	Permanence (%)	Total
Tyr124	OH	N1	16.52	39.88
	OH	N2	23.36	
Gln291	NE2	N3	0.57	10.83
	O	N4	10.26	
Ser293	N	N3	0.28	55.68
	O	N4	55.84	
Phe295	N	O1	86.61	86.61
Arg296	N	O1	21.37	21.37
Tyr341	OH	N1	4.27	10.53
	OH	N2	0.85	
	O	N4	5.41	
BuChE				
Asp70	OD1	N4	37.92	75.17
	OD2	N4	37.25	
Glu197	OE1	N2	12.42	15.08
	OE2	N2	2.66	
Tyr332	OH	N4	2.88	44.12
	OH	O1	0.22	
	OH	N1	2.66	
	OH	N2	38.36	
Lis339	NZ	N3	33.04	33.04

## Data Availability

Data is contained within the article.
